# Evaluation of Blood Biochemical Parameters and Ratios in Piroplasmosis-Infected Horses in an Endemic Region

**DOI:** 10.3390/vetsci12070643

**Published:** 2025-07-05

**Authors:** Juan Duaso, Alejandro Perez-Ecija, Ana Navarro, Esther Martínez, Adelaida De Las Heras, Francisco J. Mendoza

**Affiliations:** 1 Department of Animal Medicine and Surgery, University of Cordoba, 14014 Cordoba, Spain; juanduaso@gmail.com (J.D.); v82hesaa@uco.es (A.D.L.H.); fjmendoza@uco.es (F.J.M.); 2 Gasset Laboratory, DAV SALUD SL, 18200 Granada, Spain; ana@grupodavsalud.es (A.N.); esther@grupodavsalud.es (E.M.)

**Keywords:** *Babesia caballi*, biochemistry, inflammatory response, piroplasmosis diagnosis, *Theileria equi*

## Abstract

Equine piroplasmosis (EP), caused by *Theileria equi* and *Babesia caballi*, is a worldwide tick-borne disease with severe economic, commercial, and sanitary implications for equids. Although diagnosis is based on direct (blood smear or PCR) or indirect (serology) methods, these techniques are expensive, laborious, and false-negative and false-positive results can be yielded. Biochemistry blood profiles are routinely performed in horses. Biochemical parameters and ratios could be a reliable complementary diagnostic tool to assist clinicians in EP diagnosis, mainly in endemic areas, or for discarding similar disorders (piro-like diseases) and prioritizing specific diagnostic testing. This study describes the changes induced by EP infection in blood biochemical parameters and common and novel biochemical ratios in horses. EP-infected horses showed increased serum total and indirect bilirubin, triglycerides, and GLDH concentrations and decreased sodium concentrations compared to non-infected animals. These findings could be linked to hemolysis, diminution of athletic performance, and liver inflammation due to oxidative stress damage. While molecular methods remain the gold standard for EP diagnosis, a complete biochemical profile and ratios could provide valuable complementary information to enhance the diagnostic accuracy of piroplasmosis in horses.

## Simple Summary

Equine piroplasmosis (EP) is a tick-borne disease affecting equids (horses, donkeys, and mules) worldwide. This parasitic disorder has important health and economic impacts on the equid industry. Diagnosis is reached either by direct detection of the parasites (*Babesia caballi*, *Theileria equi*, or *Theileria haneyi*) in the bloodstream (PCR or blood smear) or by indirect methods (serology). However, it is unknown if other simpler and faster techniques, such as a biochemical profile, could help clinicians to identify this disease. In this study we describe biochemical differences between non-infected and EP-infected horses and evaluate the ability of these biochemical parameters and ratios to predict EP status.

## 1. Introduction

Equine piroplasmosis (EP) is a tick-borne disease with a worldwide distribution caused by hemoprotozoans of the genus Apicomplexa (*Theileria equi*, *Theileria haneyi*, and *Babesia caballi*) and transmitted by hard ticks belonging to the genus Ixodes [1]. EP is linked to severe economic losses in the equid industry, not only due to deaths, abortions, and veterinary costs but also because the international movement of positive animals is restricted, limiting sales and the participation of these horses in sport and show events, auctions, etc. [2,3].

EP clinical signs are variable, ranging from sudden death in hyperacute forms in non-endemic countries to milder unspecific signs such as malaise, weight loss, and decreased performance in chronic forms or even lack of evident clinical signs in the asymptomatic carrier form [4,5]. In addition, clinical signs can overlap among infections caused by *B. caballi*, *T. equi*, and *T. haneyi* [6] and even with other pathologies known as piro-like disorders such as equine anaplasmosis or leptospirosis [3].

Since clinical signs are not helpful to diagnose EP, the diagnosis is currently based on direct (blood smear, PCR) or indirect (serology) detection of the causative agents [7]. However, each of these techniques can yield false-negative or false-positive results, impairing the accuracy of EP diagnosis [3]. Since blood biochemical analyses are routinely performed in horses, they could be a reliable, fast, easy-to-perform, and cost-effective alternative diagnostic method that, in combination with the gold standard methods (PCR and serology), could improve the accuracy of EP diagnosis and help to differentiate between clinical forms and conditions caused by different parasites. Although some studies have previously evaluated the effect of EP on biochemical blood parameters [8,9,10], these reports present some limitations. In brief, a whole biochemical panel was not performed, a low number of horses were included, samples were collected from horses living in small geographic regions, and the animals were selected based on their clinical presentation (acute forms with clinical signs or chronic carrier without signs). Lastly, data were not analyzed depending on the causative agent, and only direct detection of the parasites in blood smears was used as a confirmatory EP diagnosis.

Hematologic and biochemical blood ratios are emerging biomarkers in human medicine useful as predictive diagnostic tools of several diseases (cancer, cirrhosis, sepsis, COVID-19) [11,12,13,14]. Scarce information on these ratios is available in horses, mostly focused on the suitability of hematologic ratios to determine sepsis severity and outcome [15,16]. Interestingly, a recent study demonstrated significant differences in hematologic ratios between EP-negative horses and those infected by *B. caballi*, with platelet-related ratios being deemed as the most useful to predict EP status [17]. Since EP infection triggers a systemic inflammatory response with variable intensity depending on the clinical form [18], it could be hypothesized that biochemical biomarkers could help in EP diagnosis. To the authors’ knowledge, blood biochemical ratios have not been evaluated in EP-infected horses.

The aims of this study were the following: (a) to characterize the biochemical changes induced by infection with *B. caballi* or *T. equi* in horses attending to the diagnostic method; (b) to study variations in biochemical ratios induced by EP infection; and (c) to evaluate the reliability of biochemical biomarkers and ratios to predict EP infection according to the diagnostic method.

## 2. Materials and Methods

### 2.1. Animals Inclusion Criteria and Study Design

Data from horse blood samples submitted for EP analysis to a national private veterinary reference laboratory (Gasset Laboratory, DAV Salud Group SL, Granada, Spain) during a three-year period (from January 2022 to December 2024) were retrospectively retrieved. Blood samples were shipped overnight, and biochemical analyses were performed at arrival (less than 24 h). If serology or DNA extraction was carried out the day after, samples were kept in refrigeration. Samples with lipemia, hemolysis, or any abnormal sample processing (e.g., shipping delays longer than 24 h, long storage, clotted sample, etc.) were discarded. Repeated analyses from the same horse were removed. Samples from horses co-infected with both parasites were discarded, as well as those samples from donkeys and mules. Although no information on clinical signs was included in the statistical analysis in order to avoid any bias, signalment data such as breed, sex, and age were retrieved for population description. Only analyses for *B. caballi* and *T. equi* were performed, since the presence of *T. haneyi* has not been demonstrated in Spain.

The minimum number of blood samples needed for this study (1066) was calculated using the formula for a finite population, taking into consideration the number of horses registered in Spain in 2022 (722,158) [19], a 95% of confidence interval (95% CI), and 3% of maximum error.

The following inclusion criteria were used: results from a complete advanced biochemical panel, together with EP diagnosis results, and either a real-time PCR or a serological technique (cELISA or IFAT). Firstly, in order to evaluate the effect of EP infection on biochemical parameters and ratios, horses were classified as non-EP-infected (control group, PCR− S−), PCR-positive horses (PCR+ S−), and seropositive horses (PCR− S+). Lastly, to concretely study the effect of infection by *B. caballi* or *T. equi* on biochemical parameters and ratios according to the diagnostic method used, horses were grouped into the following groups: horses PCR-negative for both parasites (T− B−), *B. caballi* PCR-positive (B+ T−), and *T. equi* PCR-positive (B− T+); and for serology in the following groups: horses seronegative for both parasites (sB− sT−), *B. caballi* seropositive (sB+ sT−), and *T. equi* seropositive horses (sB− sT+).

### 2.2. Biochemical Analysis

Submitted serum tubes were centrifugated for 10 min at 3500 rpm and analyzed for a complete biochemical panel (Spin 640 Plus, Spinreact, Barcelona, Spain) and electrolyte concentrations (e-Spin 10, Spinreact, Barcelona, Spain). The following biochemical parameters were analyzed: glucose (GLU), triglycerides (TGL), urea (URE), creatinine (CREA), total protein (TP), albumin (ALB), total bilirubin (TB), direct (conjugated) bilirubin (DB), alkaline phosphatase (ALP), gamma-glutamyl transferase (GGT), glutamate dehydrogenase (GLDH), bile acids (AB), aspartate aminotransferase (AST), creatine kinase (CK), and lactate dehydrogenase (LDH).

The electrolyte concentrations measured were sodium (Na), potassium (K), chloride (Cl), total calcium (Ca), phosphorus (P), total magnesium (Mg), and iron (Fe).

Fibrinogen (FIB) concentrations were determined using the Millar’s technique [20]. Serum symmetric dimethylarginine (SDMA) concentrations were determined using a commercial validated ELISA kit (DLD Diagnostika GmbH, Hamburg, Germany) [21]. The following parameters were calculated according to previously reported formulas: indirect (unconjugated) bilirubin (IB) and globulin concentrations (GLO).

The biochemical ratios included were the following: direct bilirubin to total bilirubin ratio (DB:TB), albumin to globulin ratio (A:G), urea to creatinine ratio (Ure:Crea), creatinine to urea ratio (Crea:Ure), urea to albumin ratio (Ure:Alb), and LDH to albumin ratio (LDH:alb) [13,14].

### 2.3. Indirect (Serological) Detection

#### 2.3.1. cELISA

Serum samples were analyzed using commercial cELISA kits (VMRD Inc., Pullman, WA, USA) previously validated in horses [22,23]. Use of these kits was performed by the same operator following protocols from the manufacturer, and optical densities were read at 630 nm in a microplate reader (ChroMate 4300, Awareness Technology Inc., Palm City, FL, USA). According to the manufacturer, these kits have a sensitivity of 95% and a specificity of 99.5% for *T. equi* and a sensitivity of 100% and a specificity of 100% for *B. caballi*.

#### 2.3.2. IFAT

Serum samples were analyzed using commercial kits (MegaFLUO^®^ *T. equi* or *B. caballi*, Megacor Diagnostik, Hörbranz, Austria) previously validated in horses [24,25]. Measurements were performed according to the manufacturer’s instructions and by the same operator. Fluorescence was detected with a trinocular fluorescence microscope (Balea 8100, Beortek SA, Amended, Spain). The exact sensitivity and specificity of these IFAT tests are unknown, but this technique is widely considered as a highly specific confirmatory test [24].

### 2.4. Direct Detection

An automatized nucleic acid extraction system (MagNA Pure 24 Instruments, Roche Diagnostics, Barcelona, Spain) was used to extract DNA from a 200 µL aliquot of EDTA samples following the manufacturer’s instructions. Extracted DNA was frozen at −20 °C until analysis. A real-time PCR was performed using commercial DNA T. equi or B. caballi detection kits (genetic PCR solutions, Alicante, Spain) and an automatized thermocycler (LightCycler 480, Roche Diagnostics, Barcelona, Spain) [17]. These kits contain individual ready-to-use tubes with all the components needed (mastermix, primers, DNase/RNase free water, etc.) to perform the PCR. Internal, negative, and positive controls were provided by the manufacturer in the same kits. PCR conditions, in a final reaction volume of 20 µL (5 µL of DNA), were as follows: initial activation at 95 °C for 2 min, followed by 40 cycles of 5 s at 95 °C (denaturation), and a final hybridization/extension step at 60 °C for 20 s.

### 2.5. Statistical Analysis

Normality was assessed using the Kolmogorov–Smirnov test, and results are expressed as mean ± standard deviation (SD) or median and interquartile range (IQR = 75th–25th percentiles) according to the distribution. The Tukey’s Hinges test was used to calculate the median and percentiles. Groups were compared using a Kruskal–Wallis test with Dunn’s post hoc test or a one-way ANOVA with Tukey’ post hoc test, depending on normality.

The ability of different biochemical parameters and ratios to predict EP status was studied using the area under the curve (AUC) in the receiver operating characteristic (ROC) curve analysis. AUCs were classified as excellent (AUC between 1.0 and 0.9); good (0.9–0.8); fair (0.8–0.7); poor (0.7–0.6); and fail (<0.6). Sensitivity and specificity for each parameter and ratio were determined using the cut-off value with higher likelihood ratio to differentiate amongst positive and negative animals. Accuracy and positive and negative predictive values were calculated based on those sensitivities and specificities.

All analyses were conducted using specific statistical packages (IBM SPSS Statistics 27, IBM Corporation, Armonk, NY, USA; and GraphPad Prism 9, San Diego, CA, USA). Values with *p* < 0.05 were considered significant.

## 3. Results

During the inclusion period, a total of 1121 horse blood samples were submitted to the laboratory for EP analysis, with 11.1% corresponding to 2022, 46.5% to 2023, and 42.5% to 2024. The mean age was 5 (4) years old (ranging from 1 to 26 years old). Regarding the gender, 24% were mares and 76% were males. The most common breed was Andalusian (69.3%), followed by crossbred (18.4%), Lusitano breed (4.5%), Spanish Sport Horse breed (CDE, 1.3%), KWPN (0.8%), Hispano-Breton breed (0.8%), Catalan Pyrenean Horse breed (0.5%), Arabian breed (0.5%), Anglo-Arabian breed (0.5%), Hispano-Arabian breed (0.5%), and other less represented breeds (<0.5%) such as Argentine Polo, Frisian, Westphalian, French Trotter, Poni crossbred, Burguete, Zangersheide, Belgian Warmblood, etc.

### 3.1. Differences in Biochemical Parameters and Ratios According to Diagnostic Method

Biochemical results for non-EP-infected horses (control group, n = 190), PCR-positive (n = 55) horses, and seropositive (n = 29) horses are compiled in Table 1. Horses in the PCR+ S− group had significantly (*p* < 0.05) lower serum CK concentrations but higher (*p* < 0.05) TGL concentrations than the control group (Table 1). No differences were observed between the control group and the serologically positive group. When both diagnostic methods were compared, PCR-positive horses showed lower (*p* < 0.05) AST concentrations than serologically positive horses (Table 1). No significant differences were observed between any groups studied for any of the ratios evaluated (Table 2).

### 3.2. Differences in Biochemical Parameters and Ratios According to the EP Status in PCR-Diagnosed Horses

Biochemical results for non-EP-infected horses (B− T−, n = 109), *B. caballi* PCR-positive horses (B+ T−, n = 11), and *T. equi* PCR-positive horses (B− T+, n = 39) are compiled in Table 3. PCR-positive horses for *B. caballi* had significantly (*p* < 0.05) higher serum TGL, TB, and IB concentrations than the control group (B− T−) but lower (*p* < 0.05) sodium concentrations (Table 3). *B. caballi*-positive horses had significantly (*p* < 0.05) higher TB and IB but lower (*p* < 0.05) Na and Cl compared to *T. equi*-infected horses (Table 3). Regarding the biochemical ratios, horses with a positive PCR result for *B. caballi* had a significantly lower (*p* < 0.05) CREA/URE ratio than the control group and A:G ratio compared to *T. equi*-positive horses (Table 4).

### 3.3. Differences in Biochemical Parameters and Ratios According to the EP Status in Serologically Diagnosed Horses

Biochemical results for horses seronegative for both parasites (sB− sT−, n = 96), *B. caballi* seropositive horses (sB+ sT−, n = 8), and *T. equi* seropositive horses (sB− sT+, n = 20) are compiled in Table 5. *B. caballi* seropositive horses had significantly (*p* < 0.05) lower serum CK concentrations compared to the control group (Table 5). *T. equi* seropositive horses showed higher (*p* < 0.05) serum TB and K concentrations than the control group and higher GLDH concentrations than sB+ sT− (*p* = 0.07) and the control group (*p* = 0.03), respectively (Table 5). In addition, sB− sT+ also had lower (*p* < 0.05) values for the DB/TB ratio compared to *B. caballi* seropositive horses (Table 6). 

### 3.4. Predictive Values of Biochemical Parameters and Ratios for EP Status

Based on the AUC results in the ROC analysis, sodium showed a good ability to predict the PCR status against *B. caballi* (AUC 0.84, *p* < 0.01), while the following parameters showed a fair ability: TGL (AUC 0.75, *p* < 0.01), TB (AUC 0.72, *p* < 0.05), and IB (AUC 0.79, *p* < 0.05) (Table S1). Analytical accuracy, sensitivity, and specificity for these ratios and parameters were moderate to good, with an overall high negative predictive value (Table S1). In contrast, no parameter was found to accurately predict a positive PCR result for *T. equi* (Table S2).

Concerning serology results, only CK (AUC 0.70, *p* < 0.05) showed a fair ability to predict a seropositive horse for *B. caballi* (Table S3), with a moderate specificity and negative predictive value. No parameter had a good or fair ability to predict a seropositive animal for *T. equi* (Table S4).

## 4. Discussion

This study evaluates the changes induced by EP infection in blood biochemical parameters and ratios depending on the diagnostic method. In addition, the reliability of these parameters to predict EP infection in horses was evaluated. Although the effects of EP on blood biochemical parameters has been previously reported in horses [8,9,26], to the authors knowledge, this is the first study evaluating biochemical ratios and several specific renal and liver biomarkers such as SDMA, bile acids, and GLDH in EP-infected horses.

PCR-positive horses for *B. caballi* had higher serum TB and IB concentrations compared to non-EP and *T. equi*-infected horses. Previous studies have also reported an increase in TB in EP-infected horses [8,9,27,28]. However, only one author specifically determined serum IB concentrations in EP-infected horses [27,28]. Our results are in consonance with these previous reports [27,28], with PCR-positive horses showing higher IB concentrations than control ones, confirming that TB was elevated due to the parasites-induced hemolysis and discarding any possible effect of an increased DB secondary to hepatic or post-hepatic disorders [29,30]. On the other hand, neither *B. caballi* nor *T. equi* seropositive horses showed significant differences in TB (or IB) compared to the control group in our study. This finding is similar to previous reports, where TB variations were absent in carriers or clinically ill horses, independently of the parasite and the diagnostic method (PCR or cELISA) [10,31].

This discrepancy could be explained by differences between clinical forms and the diagnostic method used, since some techniques are preferentially used in specific clinical stages. For example, horses with an acute clinical presentation (associated with evident clinical signs) are usually tested using PCR, whereas cELISA (serology) is more commonly used for diagnosing chronic forms. Similar findings were recently reported when hematology variations were studied in EP, with PCR-positive horses showing more significant erythrocytic variations than the control group [17]. However, due to the retrospective design of this study, clinical signs were not collected nor included in the statistical analysis; thus, this statement cannot be confirmed.

While hemolysis is the main cause of increased serum IB concentrations in horses, indirect hyperbilirubinemia and jaundice have also been described in anorectic horses, secondary to malaise or fever [30]. Anorexia induces indirect hyperbilirubinemia due to a decrease in ligandin stores, which impairs unconjugated bilirubin uptake by hepatocytes. *B. caballi* PCR-positive horses also displayed higher serum TGL concentrations (hyperlipemia) than the control group. Since hyperlipemia is an indicator of lipolysis and fat mobilization secondary to anorexia [32], it could be concluded that anorexia, along with the hemolysis primarily caused by the parasite, could be linked to the increase in IB (and subsequently in TB) in *B. caballi*-infected horses.

Inflammatory interleukins also trigger fat mobilization and subsequent hyperlipemia [32]. Nonetheless, neither fibrinogen nor iron concentrations, which are commonly used as positive and negative inflammatory markers, respectively, in horses [33], were different between groups in our study.

Thus, our results disregard the idea lipolysis activation and hyperlipemia in EP-infected horses being due to severe inflammation. Similar findings were reported in a previous study, where no significant changes were found in acute-phase proteins (haptoglobin, C-reactive protein, serum A amyloid) concentrations in *T. equi*-infected horses with a wide range of clinical signs [18]. Since pro-inflammatory interleukins were not determined in our study, future ones should evaluate them to completely confirm this assumption. Furthermore, our study is the first one describing the effect of *B. caballi* and *T. equi* infections on fibrinogen concentrations in a large cohort of EP horses, since this parameter has only been assessed in a couple of case reports [34,35].

In addition, no differences in serum globulin concentrations were observed between groups in our study. The effect of EP infection on globulin concentrations is currently a controversial topic, with some studies reporting no changes in subclinical affected horses [9] and others showing hyperglobulinemia in asymptomatic PCR- or seropositive horses [27,28,31] or in horses with clinical signs [9]. Regarding total protein and albumin concentrations, similar to previous studies [8,27,28,31], no changes were observed in our study.

Intravascular hemolysis (red blood cell breakdown inside the bloodstream) leads to increased serum phosphorus and potassium concentrations [36,37]. Since no differences were observed in these parameters compared to the control group, it can be assumed that hemolysis was mainly an extravascular event. Similar findings were observed in previous studies in clinical and subclinical EP-infected horses [9,26]. In contrast, hyperphosphatemia was observed in EP PCR-positive asymptomatic carriers in another study [31]. To the authors’ knowledge, this is the first study evaluating potassium concentrations in a large cohort of EP horses using PCR and serology and differentiating between *B. caballi* and *T. equi*.

Along with the absence of changes in direct bilirubin, the lack of any increase in specific (GGT, BA, and GLDH) or unspecific (ALP, AST, LDH) liver parameters in EP-infected horses compared to the control group could discard any hepatic or post-hepatic disorder in this condition in our study.

Since this study is the first one evaluating serum GLDH and BA concentrations in EP-infected horses, we cannot compare our results with previous studies. We did find significantly higher GLDH and TB in *T. equi* seropositive horses compared to *B. caballi* seropositive ones. This finding could be linked to liver damage secondary to chronic oxidative stress in these horses. Changes in the concentrations of oxidative stress parameters (catalase activity and paraoxonase activity, among others) have been previously reported in naturally *T. equi*-infected horses [38]. Oxidative stress also increases erythrocytic osmotic fragility and causes hemolysis [38], which could also have contributed to the higher TB observed in these horses.

Subclinical *T. equi* infection has been linked to poor performance and acute exertional rhabdomyolysis [39]. Only a slight increase in AST was observed in *EP* seropositive horses in our study, with *B. caballi*-infected horses showing lower serum CK concentrations than other groups. Similar AST findings in *T. equi*-infected horses diagnosed by PCR [27,28] and serology [9,31] and similar CK results have been previously described [8,31]. In contrast, in clinically EP-affected horses, AST and CK concentrations were increased in other studies [8,40].

At this moment it is unknown whether the commonly observed azotemia in acute EP forms is due to a direct insult to the kidney by piroplasms or is secondary either to hemoconcentration (prerenal kidney disease) [4] or to the deposit of immunocomplexes in the glomeruli (immune-mediated acute renal failure) [35]. This study is the first one evaluating serum SDMA concentrations, a specific parameter of early renal damage [41], in EP-infected horses. No changes in serum urea, Crea, or SDMA concentrations were observed in our study, which could demonstrate that this azotemia is more likely secondary and linked to severity of disease [8,9,35]. In this sense, azotemia was not observed in chronically EP asymptomatic horses in a previous report [31].

No differences in serum magnesium concentrations were observed in our study. Since no previous reports evaluating this electrolyte in EP-infected horses are available, no comparison can be made.

Several factors could explain the differences between our results and those previously reported by other authors [8,9,26,28,31,42]. Briefly, dissimilarities in the analyzers and reagents used in each study and incomplete biochemical profiles in previous studies limit the ability to compare results. Moreover, inclusion criteria are widely different, with some studies basing their EP diagnosis only on direct visualization of parasites in stained blood smears or without differentiating among *T. equi* or *B. caballi* and other reports only including asymptomatic horses, animals with acute EP clinical signs, exclusively horses dedicated to some sport activity or belonging to a specific breed, etc. Finally, factors influencing blood sample processing (i.e., anticoagulant, platelet aggregation, time until measurement, etc.) and sampling of only small geographic regions could also contribute to these discrepancies.

To the authors’ knowledge, A/G and URE/CREA ratios are the only ones previously evaluated in EP-infected horses. In our study, *B. caballi* PCR-positive horses had a low A/G ratio, similar to a previous study, although no distinction between both parasites was performed in that report [31]. Moreover, *B. caballi* PCR-positive horses also had a lower CREA/URE ratio and higher URE/CREA ratios than the control group. Azotemia was not observed in any group in our study, but early or minor changes in the glomerular filtration rate cannot be detected by serum urea or creatinine concentrations in horses [43]. In human medicine, high URE/CREA ratios have been linked to increased mortality and worst prognosis in critically ill patients independently of normal creatinine [44]. Furthermore, an increased URE/CREA ratio has been observed in *T. equi* PCR-positive horses with serum urea and creatinine concentrations within reference ranges [42]. *B. caballi* PCR-positive horses showed lower serum sodium concentrations than other groups. Hyponatremia has been previously reported in an azotemic *B. caballi*-infected pony and in a draft Breton gelding [35,45], but dehydration and azotemia were not observed in our study in any group. Interestingly, hypernatremia was observed in another dehydrated and azotemic horse, but in this case it was infected by *T. equi* [34]. Hyponatremia has been previously reported in non-azotemic *B. canis*-infected dogs with a syndrome of inappropriate antidiuretic hormone secretion (SIADH) [46]. Both hyponatremia and a decreased ratio could be explained by impairment of the renin–angiotensin–aldosterone axis and ADH secretion in *B. caballi*-infected horses. Further studies evaluating these parameters could clarify this hypothesis.

On the other hand, *T. equi* seropositive horses presented lower DB/TB ratios. In human medicine, this ratio has been associated with a poor prognosis and a more urgent need for liver transplantations [47]. In our study, although this group also had higher serum GLDH concentrations, no changes in other liver parameters were observed. Thus, while hepatitis cannot be diagnosed in these horses, the combination of high GLDH and a low DB/TB ratio could be a useful biomarker to detect *T. equi* seropositive horses needing further diagnostic methods. This finding could be due to the higher tendency of *T. equi* for liver sequestration, inducing persistent infection and impacting liver function [48].

Our third objective was to study the ability of the biochemical parameters and ratios to predict *B. caballi* or *T. equi* infection in horses. Firstly, it is paramount to clarify that an EP diagnosis should always rely on PCR or serology tests, which are the current gold standard diagnostic methods. However, if any of these parameters (which are routinely measured in horses, easy and rapid to determine, and cost-effective) can help to discard (or suspect) EP infection, clinicians can prioritize further specific tests or avoid unnecessary ones. In order to determine this ability to predict EP status, and although we did find several significant differences between groups, an ROC curve study was determined to be the best method [49]. No biochemical parameter or ratio was found to accurately predict *T. equi* positive results (neither in PCR nor in serology). According to our results, low sodium concentrations and high TGL, TB, and IB concentrations were good to fair predictors of a positive *B. caballi* PCR results, with high specificity and predictive negative value. On the other hand, only CK activity was a fair predictor of a *B. caballi* seropositive result, with a moderate specificity and predictive negative value. Since these findings are not pathognomonic for EP, it is important to note that the isolated use of biochemistry to discard/confirm *B. caballi* infection could be very risky and should not be endorsed.

Finally, it is important to highlight some weaknesses of this study. First, groups in this study were heterogeneous since they were created based on blood samples submitted to our laboratory. Moreover, the number of animals infected with *B. caballi* or *T. equi* included in each group is not homogenous, since the prevalence for *T. equi* is higher in Spain compared to *B. caballi*. Moreover, another limitation of our study is the retrospective study design, where missing data regarding background history or the reason to perform molecular or serological testing are unknown; thus, further investigation cannot be carried out due to missing anamnesis. In addition, although statistical differences were observed, most biochemical parameters studied were within the reference ranges established by our laboratory, except for TGL, TB, and IB in *B. caballi* PCR-positive horses. Finally, other piro-like infectious diseases such as anaplasmosis or leptospirosis were not evaluated in this study; thus, these co-infections could have altered some of our results.

## 5. Conclusions

Blood biochemical parameters, mostly TB, IB, GLDH, CK, AST, and sodium, and the ratios DB/TB, CREA/URE, and A/G, could be alternative complementary tools for EP diagnosis and could contribute to differentiating between *B. caballi* and *T. equi* infection. Nonetheless, further studies evaluating advanced biochemical parameters linked to muscle damage, oxidative stress, and inflammatory markers could provide valuable information for EP diagnosis, mainly as a screening tool in large farms residing in endemic regions.

## Figures and Tables

**Table 1:** Biochemical parameters (n = 274) in non-EP-infected and seronegative horses (control group, n = 190), EP PCR-positive horses (n = 55), and EP seropositive horses (n = 29).

	GLU (mmol/L)	TGL (mmol/L)	URE (mmol/L)	CREA (µmol/L)	SDMA (µmol/L)	TPs (g/L)	ALB (g/L)	GLO (g/L)	FIB (g/L)	TB (µmol/L)	DB (µmol/L)	IB (µmol/L)	ALP (IU/L)	GGT (IU/L)	GLDH (IU/L)	BAs (μmol/L)	AST (IU/L)	CK (IU/L)	LDH (IU/L)	Na (mmol/L)	K (mmol/L)	Cl (mmol/L)	Ca (mmol/L)	P (mmol/L)	Mg (mmol/L)	Fe (µmol/L)
**PCR− S−**	4.8 (2.1)	0.4 (0.3)	5.2 (2.0)	115 (44)	0.1 (0.06)	68 ± 1.0	36 (7)	31 (10)	3.0 (3.0)	36 (20)	5.1 (3.4)	27 (19)	455 (251)	16.1 (10.7)	3.5 (5.7)	5.5 (5.6)	231 (111)	216 (114)	585 (465)	135 (7)	4.2 (0.9)	100 (5)	2.9 (0.4)	1.0 (0.5)	0.8 (0.2)	26.3 ± 0.9
**PCR+ S−**	4.9 (2.2)	0.5 (1.0) ^a^	5.2 (2.5)	115 (44)	0.1 (0.06)	68 ± 1.0	35 (6)	32 (9)	3.0 (2.0)	36 (53)	5.1 (6.8)	34 (65)	500 (261)	17.2 (11.6)	3.8 (7.1)	5.1 (3.7)	203 (158)	169 (137) ^a^	585 (509)	134 (8)	4.4 (1.0)	99 (5)	2.9 (0.5)	0.9 (0.6)	0.8 (0.2)	25.4 ± 2.3
**PCR− S+**	4.5 (2.2) ^b^	0.4 (0.4)	5.6 (2.0)	115 (26)	0.08 (0.03)	67 ± 1.0	36 (7)	31 (9)	3.0 (2.2)	32 (32)	5.1 (3.4)	29 (26)	392 (328)	15.0 (8.7)	3.9 (6.1)	6.7 (6.8)	237 (116) ^b^	206 (122)	686 (431)	135 (8)	4.1 (1.3)	99 (6)	3.0 (0.4)	1.1 (0.7)	0.8 (0.2)	27.8 ± 2.2
**Reference** range *	4.4–6.1	0.2–0.6	2.5–6.7	71–177	0–1.4	55–75	25–40	20–40	1.0–4.0	5–43	1.7–8.5	3–34	60–550	5–35	0–12	0–15	100–350	50–350	0–700	133–150	3–5	95–105	2.6–3.4	0.6–1.8	0.7–1.0	14–42

Data are expressed as median (IQR, interquartile range) or mean ± standard error (SE) according to distribution. ALB, albumin; ALP, alkaline phosphatase; AST, aspartate aminotransferase; BAs, bile acids; Ca, total calcium; CK, creatine kinase; Cl, chloride; CREA, creatinine; DB, direct bilirubin; EP, equine piroplasmosis; Fe, iron; FIB, fibrinogen; GGT, gamma-glutamyl transferase; GLDH, glutamate dehydrogenase; GLO, globulin; GLU, glucose; IB, indirect bilirubin; K, potassium; LDH, lactate dehydrogenase; Mg, total magnesium; Na, sodium; P, phosphorus; SDMA, symmetric dimethylarginine; TB, total bilirubin; TGL, triglycerides; TPs, total proteins; URE, urea. ^a^ *p* < 0.05 versus control group; ^b^ *p* < 0.05 versus PCR+ S−. * Reference ranges established internally in our laboratory.

**Table 2:** Biochemical biomarkers (n = 274) in non-EP-infected and seronegative horses (control group, n = 190), EP PCR-positive horses (PCR+ S−, n = 55), and EP seropositive horses (PCR− S+, n = 29).

	ALB/GLO	DB/TB	URE/CREA	CREA/URE	URE/ALB	LDH/ALB
**PCR− S−**	1.1 ± 0.02	3.4 (1.7)	0.04 (0.02)	23.4 (10.3)	0.14 (0.05)	16.8 (13.8)
**PCR+ S−**	1.1 ± 0.04	3.4 (3.4)	0.05 (0.03)	21.8 (12.0)	0.14 (0.07)	18.3 (14.2)
**PCR− S+**	1.1 ± 0.06	3.4 (3.4)	0.05 (0.03)	19.3 (11.5)	0.16 (0.07)	20.1 (11.0)

Data are expressed as median (IQR, interquartile range). ALB/GLO, albumin to globulin ratio; URE/ALB, urea to albumin; URE/CREA, urea to creatinine ratio; CREA/URE, creatinine to urea ratio; DB/TB, direct bilirubin to total bilirubin ratio; EP, equine piroplasmosis; LDH/ALB, LDH to albumin ratio.

**Table 3:** Biochemical parameters (n = 159) in non-EP-infected horses (control group, n = 109), *B. caballi* PCR-positive horses (B+ T−, n = 11), and *T. equi* PCR-positive horses (B− T+, n = 39).

	GLU (mmol/L)	TGL (mmol/L)	URE mmol/L)	CREA (µmol/L)	SDMA (µmol/L)	TPs (g/L)	ALB (g/L)	GLO (g/L)	FIB (g/L)	TB (µmol/L)	DB (µmol/L)	IB (µmol/L)	ALP (IU/L)	GGT (IU/L)	GLDH (IU/L)	BAs (μmol/L)	AST (IU/L)	CK (IU/L)	LDH (IU/L)	Na (mmol/L)	K (mmol/L)	Cl (mmol/L)	Ca (mmol/L)	P (mmol/L)	Mg (mmol/L)	Fe (µmol/L)
**B− T−**	4.7 (2.1)	0.4 (0.3)	5.0 (1.8)	115 (36)	0.09 (0.05)	68 ± 1.0	36 (6)	31 (10)	3.0 (3.0)	37 (29)	5.1 (3.4)	31 (22)	458 (265)	16.3 (9.0)	4.0 (6.1)	5.3 (6.1)	227 (106)	214 (126)	536 (514)	135 (7)	4.2 (0.7)	99 (5)	2.9 (0.4)	1.0 (0.4)	0.8 (0.2)	25.7 ± 1.2
**B+ T−**	5.2 (2.9)	0.8 (1.7) ^a,¶^	5.5 (2.8)	97 (26)	0.12 (0.17)	66 ± 2.0	33 (13)	36 (11)	3.0 (2.5)	87 (97) ^a,¶^	6.8 (22.2)	106 (89) ^a,¶^	428 (160)	13.9 (14.0)	5.6 (7.6)	5.5 (17.7)	193 (175)	118 (233)	621 (927)	131 (3) ^a^	4.5 (1.2)	97 (3)	3.2 (0.3)	0.8 (1.1)	0.9 (0.3)	26.9 ± 6.6
**B− T+**	4.8 (1.4)	0.4 (0.8) ^a^	5.0 (2.7)	115 (53)	0.08 (0.06)	68 ± 1.0	35 (6)	31 (8)	3.0 (2.0)	31 (34) ^b^	5.1 (6.8)	26 (26) ^b^	501 (318)	19.2 (10.5)	3.4 (4.8)	5.0 (3.8)	207 (138)	175 (131)	539 (352)	137 (8) ^b^	4.4 (1.0)	100 (3) ^b^	0.7 (0.4)	0.9 (0.4)	0.8 (0.3)	27.4 ± 2.7
**Reference** range *	4.4–6.1	0.2–0.6	2.5–6.7	71–177	0–1.4	55–75	25–40	20–40	1.0–4.0	5–43	1.7–8.5	3–34	60–550	5–35	0–12	0–15	100–350	50–350	0–700	133–150	3–5	95–105	2.6–3.4	0.6–1.8	0.7–1.0	14–42

Data are expressed as median (IQR, interquartile range) or mean ± standard error (SE) according to distribution. A/G, albumin to globulin ratio; ALB, albumin; ALP, alkaline phosphatase; AST, aspartate aminotransferase; BAs, bile acids; Ca, total calcium; CK, creatine kinase; Cl, chloride; CREA, creatinine; DB, direct bilirubin; EP, equine piroplasmosis; Fe, iron; FIB, fibrinogen; GGT, gamma-glutamyl transferase; GLDH, glutamate dehydrogenase; GLO, globulin; GLU, glucose; IB, indirect bilirubin; K, potassium; LDH, lactate dehydrogenase; Mg, total magnesium; Na, sodium; P, phosphorus; SDMA, symmetric dimethylarginine; TB, total bilirubin; TGL, triglycerides; TPs, total proteins; URE, urea. ^a^ *p* < 0.05 versus control group; ^b^ *p* < 0.05 versus B+ T−. * Reference ranges established internally in our laboratory. ^¶^ Out of our reference range.

**Table 4:** Biochemical biomarkers (n = 159) in non-EP-infected horses (control group, n = 109), *B. caballi* PCR-positive horses (B+ T−, n = 11), and *T. equi* PCR-positive horses (B− T+, n = 39).

	ALB/GLO	DB/TB	URE/CREA	CREA/URE	URE/ALB	LDH/ALB
**B− T−**	1.2 ± 0.03	0.1 (0.2)	0.04 (0.02)	23.6 (9.4)	0.14 (0.05)	15.9 (14.3)
**B+ T−**	1.0 ± 0.13	0.1 (0.2)	0.05 (0.03)	19.8 (9.4) ^a^	0.17 (0.16)	18.2 (38.1)
**B− T+**	1.1 ± 0.05 ^b^	0.2 (0.1)	0.04 (0.03)	24.7 (16.2)	0.12 (0.08)	16.7 (10.9)

Data are expressed as median (IQR, interquartile range) or mean ± standard error (SE) according to distribution. ALB/GLO, albumin to globulin ratio; URE/ALB, urea to albumin; URE/CREA, urea to creatinine ratio; CREA/URE, creatinine to urea ratio; EP, equine piroplasmosis; DB/TB, direct bilirubin to total bilirubin ratio; LDH/ALB, LDH to albumin ratio. ^a^ *p* < 0.05 versus Control group; ^b^ *p* < 0.05 versus B+ T−.

**Table 5:** Biochemical parameters (n = 124) in horses seronegative for both parasites (control group [sT− sB−], n = 96), *B. caballi* seropositive horses (sB+ sT−, n = 8), and *T. equi* seropositive horses (sB− sT+, n = 20).

	GLU (mmol/L)	TGL (mmol/L)	URE (mmol/L)	CREA (µmol/L)	SDMA (µmol/L)	TPs (g/L)	ALB (g/L)	GLO (g/L)	FIB (g/L)	TB (µmol/L)	DB (µmol/L)	IB (µmol/L)	ALP (IU/L)	GGT (IU/L)	GLDH (IU/L)	BAs (μmol/L)	AST (IU/L)	CK (IU/L)	LDH (IU/L)	Na (mmol/L)	K (mmol/L)	Cl (mmol/L)	Ca (mmol/L)	P (mmol/L)	Mg (mmol/L)	Fe (μmol/L)
**sB− sT−**	4.9 (2.1)	0.35 (0.3)	5.3 (2.08)	124 (44.2)	0.10 (0.06)	67 ± 1.0	36 (7)	32 (10)	3.0 (3.0)	32 (18)	5.1 (3.4)	23 (19)	448 (299)	15.9 (13.2)	3.1 (5.1)	6.3 (5.5)	233 (109)	219 (110)	614 (399)	135 (10)	4.1 (0.9)	100 (4)	2.9 (0.3)	0.9 (0.5)	0.9 (0.1)	26 ± 1.3
**sB+ sT−**	3.9 (2.3)	0.36 (0.2)	5.7 (1.83)	106 (26.5)	0.08 (0.03)	68 ± 1.0	36 (6)	32 (10)	2.5 (6.2)	26 (15)	6.8 (1.7)	19 (27)	402 (429)	13.6 (34.4)	2.1 (2.8)	6.0 (4.7)	226 (115)	152 (156) ^a^	757 (403) ^¶^	140 (9)	3.9 (0.5)	101 (6)	2.9 (0.4)	1.0 (1.0)	0.9 (0.2)	28 ± 5.2
**sB− sT+**	4.7 (2.2)	0.46 (0.9)	5.7 (3.55)	115 (35.4)	0.08 (0.03)	67 ± 1.0	36 (9)	32 (8)	3.0 (2.5)	43 (53) ^b^	5.1 (3.4)	32 (41)	418 (457)	15.1 (12.0)	4.2 (5.7) ^a^	7.7 (7.6)	253 (133)	213 (131)	618 (662)	134 (11)	4.3 (1.8) ^b^	98 (5)	2.9 (0.5)	1.1 (0.8)	0.8 (0.3)	27 ± 2.6
**Reference** range *	4.4–6.1	0.2–0.6	2.5–6.7	71–177	0–1.4	55–75	25–40	20–40	1.0–4.0	5–43	1.7–8.5	3–34	60–550	5–35	0–12	0–15	100–350	50–350	0–700	133–150	3–5	95–105	2.6–3.4	0.6–1.8	0.7–1.0	14–42

Data are expressed as median (IQR, interquartile range) or mean ± standard error (SE) according to distribution. ALB, albumin; ALP, alkaline phosphatase; AST, aspartate aminotransferase; BAs, bile acids; Ca, total calcium; CK, creatine kinase; Cl, chloride; CREA, creatinine; DB, direct bilirubin; EP, equine piroplasmosis; Fe, iron; FIB, fibrinogen; GGT, gamma-glutamyl transferase; GLDH, glutamate dehydrogenase; GLO, globulin; GLU, glucose; IB, indirect bilirubin; K, potassium; LDH, lactate dehydrogenase; Mg, total magnesium; Na, sodium; P, phosphorus; SDMA, symmetric dimethylarginine; TB, total bilirubin; TGL, triglycerides; TPs, total proteins; URE, urea. ^a^ *p* < 0.05 versus control group; ^b^ *p* < 0.05 versus B+ T−. * Reference ranges established internally in our laboratory. ^¶^ Out of our reference range.

**Table 6:** Hematological biomarkers (n = 124) in horses seronegative for both parasites (control group [sT− sB−], n = 96), *B. caballi* seropositive horses (sB+ sT−, n = 8), and *T. equi* seropositive horses (sB− sT+, n = 20).

	ALB/GLO	DB/TB	URE/CREA	CREA/URE	URE/ALB	LDH/ALB
**sB− sT−**	1.1 ± 0.1	0.2 (0.1)	0.04 (0.03)	22.7 (12.7)	0.15 (0.07)	18.3 (10.1)
**sB+ sT−**	1.1 ± 0.1	0.3 (0.2)	0.06 (0.02)	17.8 (9.1)	0.16 (0.05)	20.7 (9.3)
**sB− sT+**	1.1 ± 0.1	0.1 (0.2) ^b^	0.05 (0.04)	20.4 (13.0)	0.16 (0.09)	16.9 (18.3)

Data are expressed as median (IQR, interquartile range) or mean ± standard error (SE) according to distribution. ALB/GLO, albumin to globulin ratio; URE/ALB, urea to albumin ratio; URE/CREA, urea to creatinine ratio; CREA/URE, creatinine to urea ratio; DB/TB, direct bilirubin to total bilirubin ratio; EP, equine piroplasmosis; LDH/ALB, LDH to albumin ratio. ^b^ *p* < 0.05 versus sB+ sT−.

## Data Availability

Data are available upon request to the corresponding author.

## Supplementary Materials

The following supporting information can be downloaded at: https://www.mdpi.com/article/10.3390/vetsci12070643/s1. Performance of biochemical parameters and ratios for predicting *B. caballi* infection by PCR (Table S1), *T. equi* infection by PCR (Table S2), *B. caballi* infection by serology (Table S3), and *T. equi* infection by serology (Table S4).
