# Supplementary material for: Evaluation of Blood Biochemical Parameters and Ratios in Piroplasmosis-Infected Horses in an Endemic Region
Source: Vet Sci. 2025 Jul 5;12(7):643. doi: 10.3390/vetsci12070643 (PMC12300670; doi:10.3390/vetsci12070643)
Supplement: Supplementary file 1 [file vetsci-12-00643-s001.zip › Table S1..pdf]

Table S1. Performance of biochemical parameters and ratios for predicting *B. caballi* infection by PCR.

Variable	AUC (95% CI)	p value	SEN (95% CI)	SPE (95% CI)	ACC	PPV	NPV
GLU	0.588 (0.371 - 0.805)	0.383	0.111 (0.005 - 0.435)	0.989 (0.941 - 0.999)	0.912	0.500	0.920
TGL	0.752 (0.611 - 0.892)	0.009	0.100 (0.005 - 0.404)	0.989 (0.942 - 0.999)	0.904	0.500	0.912
URE	0.610 (0.430 - 0.790)	0.254	0.200 (0.035 - 0.509)	0.936 (0.867 - 0.970)	0.865	0.250	0.917
CREA	0.648 (0.493 - 0.803)	0.123	0.600 (0.312 - 0.831)	0.718 (0.624 - 0.796)	0.708	0.171	0.949
SDMA	0.639 (0.355 - 0.923)	0.262	0.333 (0.059 - 0.700)	0.969 (0.893 - 0.994)	0.914	0.500	0.939
TP	0.559 (0.393 - 0.724)	0.538	0.200 (0.035 - 0.509)	0.895 (0.822 - 0.940)	0.835	0.154	0.922
ALB	0.660 (0.436 - 0.882)	0.113	0.222 (0.039 - 0.547)	0.981 (0.932 - 0.996)	0.920	0.500	0.936
GLO	0.618 (0.445 - 0.790)	0.241	0.556 (0.266 - 0.811)	0.709 (0.614 - 0.787)	0.696	0.143	0.948
FIB	0.524 (0.330 - 0.716)	0.817	0.889 (0.5650 - 0.994)	0.282 (0.197 - 0.385)	0.340	0.116	0.960
TB	0.715 (0.491 - 0.939)	0.043	0.375 (0.136 - 0.694)	0.971 (0.9178 - 0.992)	0.928	0.500	0.952
DB	0.672 (0.465 - 0.877)	0.166	0.167 (0.008 - 0.563)	0.985 (0.917 - 0.999)	0.915	0.500	0.928
IB	0.788 (0.553 - 1.000)	0.020	0.667 (0.300 - 0.940)	0.969 (0.893 - 0.994)	0.943	0.667	0.969
ALP	0.554 (0.392 - 0.716)	0.591	0.111 (0.005 - 0.435)	0.936 (0.867 - 0.970)	0.864	0.143	0.917
GGT	0.632 (0.419 - 0.844)	0.191	0.222 (0.039 - 0.547)	0.960 (0.902 - 0.984)	0.900	0.333	0.933
GLDH	0.548 (0.346 - 0.750)	0.653	0.625 (0.305 - 0.863)	0.626 (0.523 - 0.718)	0.626	0.128	0.950
BA	0.586 (0.390 - 0.781)	0.489	0.167 (0.008 - 0.563)	0.985 (0.917 - 0.999)	0.915	0.500	0.928
AST	0.581 (0.362 - 0.800)	0.419	0.111 (0.005 - 0.435)	0.981 (0.931 - 0.996)	0.911	0.333	0.927
CK	0.640 (0.409 - 0.869)	0.146	0.2009 (0.035- 0.509)	0.961 (0.903 - 0.984)	0.893	0.333	0.925
LDH	0.536 (0.314 - 0.757)	0.710	0.100 (0.005 - 0.404)	0.989 (0.941 - 0.999)	0.902	0.500	0.910
Na	0.839 (0.750 - 0.926)	0.006	1.000 (0.609 - 1.000)	0.709 (0.600 - 0.797)	0.765	0.231	1.000
K	0.520 (0.269 - 0.770)	0.871	0.667 (0.300 - 0.940)	0.608 (0.497 - 0.707)	0.600	0.111	0.959
Cl	0.677 (0.518 - 0.836)	0.122	0.857 (0.486 - 0.992)	0.570 (0.459 - 0.673)	0.605	0.114	0.941
Ca	0.657 (0.414 - 0.900)	0.201	0.667 (0.300 - 0.940)	0.800 (0.699 - 0.873)	0.779	0.190	0.969
P	0.581 (0.282 - 0.880)	0.509	0.333 (0.059 - 0.700)	0.911 (0.828 - 0.956)	0.871	0.222	0.947
Mg	0.570 (0.332 - 0.806)	0.571	0.167 (0.008 - 0.563)	0.975 (0.912 - 0.995)	0.918	0.333	0.939
Fe	0.514 (0.242 - 0.785)	0.911	0.167 (0.008 - 0.563)	0.962 (0.894 - 0.989)	0.094	0.750	0.062
A:G	0.669 (0.464 - 0.873)	0.094	0.778 (0.452 - 0.960)	0.621 (0.524 - 0.709)	0.625	0.133	0.955
DB:TB	0.612 (0.343 - 0.879)	0.369	0.667 (0.300 - 0.940)	0.692 (0.572 - 0.791)	0.690	0.167	0.957
URE:CREA	0.716 (0.578 - 0.854)	0.025	0.900 (0.595 - 0.994)	0.538 (0.436 - 0.635)	0.563	0.157	0.962
CREA:URE	0.716 (0.578 - 0.854)	0.025	0.900 (0.595 - 0.994)	0.538 (0.436 - 0.635)	0.563	0.157	0.962
URE:ALB	0.702 (0.515 - 0.888)	0.047	0.667 (0.354 - 0.879)	0.681 (0.581 - 0.766)	0.680	0.167	0.955
LDH:ALB	0.606 (0.361 - 0.850)	0.294	0.444 (0.188 - 0.733)	0.794 (0.699 - 0.863)	0.760	0.174	0.935

ACC, accuracy; A:G, albumin to globulin ratio; ALB, albumin; ALP, alkaline phosphatase; AST, aspartate aminotransferase; AUC, area under curve; BA, bile acids; URE:ALB, urea to albumin; URE:CREA, urea to creatinine ratio; Ca, total calcium; CK, creatine kinase; Cl, chloride; CI, confidence interval; CREA, creatinine; CREA:URE, creatinine to urea ratio; DB, direct bilirubin; DB:TB, direct bilirubin to total bilirubin; EP, equine piroplasmosis; Fe, iron; FIB, fibrinogen; GGT, gamma-glutamyl transferase; GLDH, glutamate dehydrogenase; GLO, globulin; GLU, glucose; IB, indirect bilirubin; K, potassium; LDH, lactate dehydrogenase; LDH:ALB, LDH to albumin; Mg, total magnesium; Na, sodium; NPV, negative predictive value; P, phosphorus; PPV, positive predictive value; SDMA, symmetric dimethylarginine; TB, total bilirubin; TGL, triglycerides; TP, total proteins; SEN, sensitivity; SPE, specificity; URE, urea.
