# Supplementary material for: Evaluation of Blood Biochemical Parameters and Ratios in Piroplasmosis-Infected Horses in an Endemic Region
Source: Vet Sci. 2025 Jul 5;12(7):643. doi: 10.3390/vetsci12070643 (PMC12300670; doi:10.3390/vetsci12070643)
Supplement: Supplementary file 1 [file vetsci-12-00643-s001.zip › Table S2..pdf]

Table S2. Performance of biochemical parameters and ratios for predicting *T. equi* infection by PCR.

Variable	AUC (95% CI)	p value	SEN (95% CI)	SPE (95% CI)	ACC	PPV	NPV
**GLU**	0.563 (0.443 - 0.68)	0.309	0.793 (0.616 - 0.901)	0.355 (0.265 - 0.456)	0.459	0.277	0.846
**TGL**	0.613 (0.493 - 0.733)	0.066	0.552 (0.375 - 0.715)	0.628 (0.526 - 0.718)	0.610	0.314	0.819
**URE**	0.535 (0.404 - 0.665)	0.566	0.233 (0.117 - 0.409)	0.947 (0.881 - 0.977)	0.774	0.583	0.795
**CREA**	0.536 (0.417 - 0.654)	0.520	0.270 (0.154 - 0.429)	0.884 (0.807 - 0.932)	0.707	0.417	0.767
**SDMA**	0.539 (0.387 - 0.690)	0.614	0.444 (0.245 - 0.662)	0.750 (0.631 - 0.839)	0.683	0.333	0.828
**TP**	0.507 (0.400 - 0.612)	0.904	0.919 0.787 - 0.972)	0.133 (0.0811 - 0.211)	0.338	0.272	0.824
**ALB**	0.522 (0.410 - 0.634)	0.687	0.541 0.383 - 0.689)	0.567 (0.471 - 0.658)	0.560	0.308	0.776
**GLO**	0.532 (0.426 - 0.638)	0.564	0.778 0.619 - 0.882)	0.379 (0.290 - 0.475)	0.482	0.304	0.830
**FIB**	0.527 (0.411 - 0.641)	0.660	0.813 (0.646 - 0.911)	0.282 (0.197 - 0.385)	0.410	0.282	0.750
**TB**	0.579 (0.461 - 0.697)	0.157	0.611 (0.448 - 0.752)	0.660 (0.564 - 0.744)	0.647	0.386	0.829
**DB**	0.619 (0.469 - 0.767)	0.143	0.688 (0.444 - 0.858)	0.523 (0.403 - 0.639)	0.556	0.262	0.872
**IB**	0.551 (0.388 - 0.713)	0.528	0.563 (0.331 - 0.769)	0.641 (0.518 - 0.747)	0.625	0.281	0.854
**ALP**	0.538 (0.416 - 0.658)	0.531	0.548 (0.377 - 0.708)	0.585 (0.484 - 0.679)	0.576	0.304	0.797
**GGT**	0.561 (0.450 - 0.670)	0.282	0.500 (0.344 - 0.655)	0.723 (0.628 - 0.800)	0.642	0.367	0.795
**GLDH**	0.542 (0.425 - 0.657)	0.507	0.500 (0.326 - 0.673)	0.648 (0.546 - 0.738)	0.613	0.304	0.808
**BA**	0.575 (0.432 - 0.718)	0.331	0.889 (0.672 - 0.980)	0.354 (0.248 - 0.475)	0.470	0.276	0.920
**AST**	0.550 (0.439 - 0.660)	0.367	0.676 (0.514 - 0.803)	0.476 (0.381 - 0.571)	0.529	0.316	0.803
**CK**	0.578 (0.467 - 0.689)	0.158	0.541 (0.383 - 0.689)	0.686 (0.590 - 0.768)	0.647	0.385	0.805
**LDH**	0.500 (0.416 - 0.583)	>0.9999	0.946 (0.879 - 0.976)	0.054 (0.0234 - 0.121)	0.242	0.223	0.500
**Na**	0.553 (0.411 - 0.693)	0.444	0.609 (0.407 - 0.778)	0.532 (0.422 - 0.637)	0.520	0.259	0.813
**K**	0.567 (0.430 - 0.704)	0.329	0.478 (0.292 - 0.670)	0.772 (0.668 - 0.850)	0.686	0.355	0.831
**Cl**	0.567 (0.435 - 0.699)	0.329	0.826 (0.628 - 0.930)	0.430 (0.326 - 0.540)	0.490	0.284	0.886
**Ca**	0.527 (0.393 - 0.659)	0.698	0.565 (0.368 - 0.743)	0.588 (0.478 - 0.688)	0.583	0.273	0.814
**P**	0.542 (0.408 - 0.674)	0.546	0.565 (0.368 - 0.743)	0.570 (0.459 - 0.673)	0.578	0.273	0.810
**Mg**	0.539 (0.393 - 0.684)	0.576	0.591 (0.387 - 0.767)	0.608 (0.497 - 0.707)	0.614	0.282	0.823
**Fe**	0.519 (0.373 - 0.665)	0.783	0.273 (0.131 - 0.481)	0.835 (0.738 - 0.901)	0.685	0.316	0.781
**A:G**	0.522 (0.417 - 0.627)	0.690	0.778 (0.619 - 0.882)	0.369 (0.282 - 0.465)	0.647	0.400	0.865
**DB:TB**	0.619 (0.477 - 0.761)	0.141	0.875 (0.639 - 0.977)	0.446 (0.331 - 0.566)	0.525	0.280	0.933
**URE:CREA**	0.522 (0.385 - 0.657)	0.727	0.483 (0.313 - 0.655)	0.656 (0.554 - 0.744)	0.615	0.304	0.803
**CREA:URE**	0.522 (0.385 - 0.657)	0.727	0.483 (0.313 - 0.655)	0.656 (0.554 - 0.744)	0.615	0.304	0.803
**URE:ALB**	0.554 (0.413 - 0.695)	0.378	0.241 (0.122 - 0.421)	0.968 (0.910 - 0.991)	0.797	0.700	0.805
**LDH:ALB**	0.517 (0.398 - 0.634)	0.789	0.138 (0.054 - 0.305)	0.978 (0.924 - 0.996)	0.783	0.800	0.783

ACC, accuracy; A:G, albumin to globulin ratio; ALB, albumin; ALP, alkaline phosphatase; AST, aspartate aminotransferase; AUC, area under curve; BA, bile acids; URE:ALB, urea to albumin; URE:CREA, urea to creatinine ratio; Ca, total calcium; CK, creatine kinase; Cl, chloride; CI, confidence interval; CREA, creatinine; CREA:URE, creatinine to urea ratio; DB, direct bilirubin; DB:TB, direct bilirubin to total bilirubin; EP, equine piroplasmosis; Fe, iron; FIB, fibrinogen; GGT, gamma-glutamyl transferase; GLDH, glutamate dehydrogenase; GLO, globulin; GLU, glucose; IB, indirect bilirubin; K, potassium; LDH, lactate dehydrogenase; LDH:ALB, LDH to albumin; Mg, total magnesium; Na, sodium; NPV, negative predictive value; P, phosphorus; PPV, positive predictive value; SDMA, symmetric dimethylarginine; TB, total bilirubin; TGL, triglycerides; TP, total proteins; SEN, sensitivity; SPE, specificity; URE, urea.
