# Supplementary material for: Evaluation of Blood Biochemical Parameters and Ratios in Piroplasmosis-Infected Horses in an Endemic Region
Source: Vet Sci. 2025 Jul 5;12(7):643. doi: 10.3390/vetsci12070643 (PMC12300670; doi:10.3390/vetsci12070643)
Supplement: Supplementary file 1 [file vetsci-12-00643-s001.zip › Table S3..pdf]

Table S3. Performance of biochemical parameters and ratios for predicting *B. caballi* infection by serology.

Variable	AUC (95% CI)	p value	SEN (95% CI)	SPE (95% CI)	ACC	PPV	NPV
**GLU**	0.606 (0.406 - 0.806)	0.321	0.625 (0.305 - 0.863)	0.644 (0.538 - 0.736)	0.642	0.139	0.949
**TGL**	0.520 (0.366 - 0.674)	0.849	1.000 (0.675 - 1.000)	0.258 (0.178 - 0.358)	0.305	0.108	1.000
**URE**	0.516 (0.322 - 0.709)	0.880	0.750 (0.409 - 0.955)	0.449 (0.350 - 0.552)	0.463	0.109	0.950
**CREA**	0.661 (0.519 - 0.802)	0.110	1.000 (0.700 - 1.000)	0.300 (0.218 - 0.395)	0.358	0.114	1.000
**SDMA**	0.677 (0.517 - 0.836)	0.307	1.000 (0.438 - 1.000)	0.583 (0.442 - 0.711)	0.608	0.130	1.000
**TP**	0.532 (0.421 - 0.642)	0.754	1.000 (0.700 - 1.000)	0.360 (0.272 - 0.457)	0.413	0.123	1.000
**ALB**	0.537 (0.363 - 0.710)	0.715	1.000 (0.700 - 1.000)	0.255 (0.179 - 0.349)	0.318	0.110	1.000
**GLO**	0.528 (0.343 - 0.713)	0.779	1.000 (0.700 - 1.000)	0.174 (0.111 - 0.260)	0.243	0.100	1.000
**FIB**	0.584 (0.240 - 0.927)	0.575	0.500 (0.088 -0.911)	0.716 (0.599 - 0.810)	0.761	0.118	0.963
**TB**	0.685 (0.510 - 0.859)	0.067	0.667 (0.354 - 0.879)	0.704 (0.607 - 0.785)	0.701	0.171	0.958
**DB**	0.629 (0.458 - 0.798)	0.459	1.000 (0.438 - 1.000)	0.458 (0.325 - 0.597)	0.490	0.103	1.000
**IB**	0.670 (0.424 - 0.916)	0.327	1.000 (0.438 - 1.000)	0.396 (0.270 - 0.536)	0.431	0.094	1.000
**ALP**	0.514 (0.286 - 0.742)	0.893	0.500 (0.215 - 0.784)	0.678 (0.574 - 0.767)	0.663	0.125	0.937
**GGT**	0.559 0.324 - 0.793)	0.559	0.444 (0.188 - 0.733)	0.804 (0.714 - 0.870)	0.774	0.174	0.940
**GLDH**	0.553 (0.399 - 0.707)	0.618	0.750 (0.409 - 0.955)	0.506 (0.403 - 0.607)	0.526	0.120	0.957
**BA**	0.611 (0.420 - 0.801)	0.522	1.000 (0.438 - 1.000)	0.521 (0.383 - 0.655)	0.549	0.115	1.000
**AST**	0.596 (0.425 - 0.767)	0.340	1.000 (0.700 - 1.000)	0.296 (0.214 - 0.392)	0.355	0.115	1.000
**CK**	0.701 (0.508 - 0.885)	0.050	0.556 (0.266 - 0.811)	0.820 (0.733 - 0.883)	0.798	0.217	0.953
**LDH**	0.641 (0.475 - 0.806)	0.189	0.750 (0.409 - 0.955)	0.580 (0.475 - 0.677)	0.594	0.140	0.962
**Na**	0.651 (0.475 - 0.827)	0.220	1.000 (0.609 - 1.000)	0.338 (0.238 - 0.453)	0.390	0.113	1.000
**K**	0.670 (0.495 - 0.844)	0.168	0.833 (0.436 - 0.991)	0.535 (0.420 - 0.646)	0.558	0.132	0.974
**Cl**	0.523 (0.278 - 0.768)	0.851	0.667 (0.300 - 0.940)	0.431 (0.322 - 0.545)	0.449	0.089	0.939
**Ca**	0.502 (0.282 - 0.722)	0.985	0.500 (0.187 - 0.812)	0.732 (0.619 - 0.821)	0.714	0.136	0.945
**P**	0.511 (0.273 - 0.747)	0.932	0.667 (0.300 - 0.940)	0.549 (0.434 - 0.659)	0.558	0.111	0.951
**Mg**	0.535 (0.298 - 0.771)	0.776	0.833 (0.436 - 0.991)	0.310 (0.214 - 0.424)	0.351	0.093	0.957
**Fe**	0.568 (0.319 - 0.816)	0.582	0.833 (0.436 - 0.991)	0.437 (0.327 - 0.552)	0.468	0.111	0.969
**A:G**	0.509 (0.291 - 0.726)	0.929	0.444 (0.188 - 0.733)	0.704 (0.607 - 0.785)	0.682	0.121	0.932
**DB:TB**	0.722 (0.514 - 0.930)	0.200	0.667 (0.118 - 0.982)	0.813 (0.680 - 0.898)	0.804	0.182	0.975
**URE:CREA**	0.639 (0.457 - 0.820)	0.194	0.750 (0.409 - 0.955)	0.584 (0.480 - 0.681)	0.598	0.140	0.963
**CREA:URE**	0.639 (0.457 - 0.820)	0.194	0.750 (0.409 - 0.955)	0.584 (0.480 - 0.681)	0.598	0.140	0.963
**URE:ALB**	0.535 (0.360 - 0.708)	0.748	0.750 (0.409 - 0.955)	0.552 (0.447 - 0.651)	0.558	0.130	0.959
**LDH:ALB**	0.642 (0.481 - 0.803)	0.184	0.625 (0.305 - 0.863)	0.663 (0.557 - 0.753)	0.660	0.147	0.950

ACC, accuracy; A:G, albumin to globulin ratio; ALB, albumin; ALP, alkaline phosphatase; AST, aspartate aminotransferase; AUC, area under curve; BA, bile acids; URE:ALB, urea to albumin; URE:CREA, urea to creatinine ratio; Ca, total calcium; CK, creatine kinase; Cl, chloride; CI, confidence interval; CREA, creatinine; CREA:URE, creatinine to urea ratio; DB, direct bilirubin; DB:TB, direct bilirubin to total bilirubin; EP, equine piroplasmosis; Fe, iron; FIB, fibrinogen; GGT, gamma-glutamyl transferase; GLDH, glutamate dehydrogenase; GLO, globulin; GLU, glucose; IB, indirect bilirubin; K, potassium; LDH, lactate dehydrogenase; LDH:ALB, LDH to albumin; Mg, total magnesium; Na, sodium; NPV, negative predictive value; P, phosphorus; PPV, positive predictive value; SDMA, symmetric dimethylarginine; TB, total bilirubin; TGL, triglycerides; TP, total proteins; SEN, sensitivity; SPE, specificity; URE, urea.
