# Supplementary material for: Evaluation of Blood Biochemical Parameters and Ratios in Piroplasmosis-Infected Horses in an Endemic Region
Source: Vet Sci. 2025 Jul 5;12(7):643. doi: 10.3390/vetsci12070643 (PMC12300670; doi:10.3390/vetsci12070643)
Supplement: Supplementary file 1 [file vetsci-12-00643-s001.zip › Table S4..pdf]

Table S4. Performance of biochemical parameters and ratios for predicting *T. equi* infection by serology.

Variable	AUC (95% CI)	p value	SEN (95% CI)	SPE (95% CI)	ACC	PPV	NPV
**GLU**	0.577 (0.424 - 0.729)	0.332	0.813 (0.569 - 0.934)	0.391 (0.295 - 0.495)	0.447	0.185	0.895
**TGL**	0.567 (0.388 - 0.746)	0.392	0.438 (0.231 - 0.668)	0.843 (0.753 - 0.903)	0.777	0.333	0.890
**URE**	0.582 (0.421 - 0.743)	0.297	0.313 (0.141 - 0.556)	0.865 (0.779 - 0.921)	0.777	0.294	0.872
**CREA**	0.531 (0.400 - 0.662)	0.660	1.000 (0.838 - 1.000)	0.100 (0.055 - 0.174)	0.250	0.182	1.000
**SDMA**	0.605 (0.431 - 0.778)	0.249	0.846 (0.577 - 0.972)	0.521 (0.383 - 0.655)	0.590	0.324	0.926
**TP**	0.502 (0.372 - 0.632)	0.975	0.667 (0.453 - 0.828)	0.400 (0.309 - 0.498)	0.446	0.189	0.851
**ALB**	0.516 (0.370 - 0.661)	0.815	0.238 (0.106 - 0.450)	0.929 (0.859 - 0.965)	0.807	0.417	0.850
**GLO**	0.527 (0.391 - 0.663)	0.704	0.750 (0.531 - 0.888)	0.439 (0.344 - 0.537)	0.492	0.214	0.896
**FIB**	0.560 (0.407 - 0.712)	0.449	0.471 (0.261 - 0.690)	0.716 (0.599 - 0.810)	0.714	0.348	0.852
**TB**	0.576 (0.426 - 0.726)	0.274	0.429 (0.244 - 0.634)	0.796 (0.705 - 0.863)	0.731	0.310	0.867
**DB**	0.519 (0.344 - 0.692)	0.833	0.429 (0.213 - 0.674)	0.667 (0.525 - 0.783)	0.613	0.273	0.800
**IB**	0.599 (0.400 - 0.796)	0.279	0.615 (0.355 - 0.822)	0.708 (0.568 - 0.817)	0.689	0.364	0.872
**ALP**	0.507 (0.342 - 0.671)	0.926	0.278 (0.125 - 0.508)	0.828 (0.734 - 0.892)	0.733	0.250	0.847
**GGT**	0.522 (0.386 - 0.657)	0.752	0.667 (0.453 - 0.828)	0.464 (0.367 - 0.562)	0.492	0.200	0.849
**GLDH**	0.626 (0.497 - 0.755)	0.093	0.833 (0.607 - 0.941)	0.494 (0.392 - 0.596)	0.551	0.250	0.936
**BA**	0.566 (0.370 - 0.761)	0.443	0.467 (0.248 - 0.698)	0.875 (0.753 - 0.941)	0.778	0.538	0.840
**AST**	0.568 (0.418 - 0.717)	0.340	0.400 (0.218 - 0.613)	0.837 (0.751 - 0.896)	0.763	0.333	0.872
**CK**	0.547 (0.401 - 0.692)	0.508	0.400 (0.218 - 0.613)	0.810 (0.722 - 0.874)	0.742	0.296	0.871
**LDH**	0.509 (0.336 - 0.680)	0.914	0.313 (0.141 - 0.556)	0.898 (0.816 - 0.945)	0.808	0.357	0.878
**Na**	0.565 (0.402 - 0.728)	0.441	0.643 (0.387 - 0.836)	0.563 (0.447 - 0.672)	0.576	0.225	0.889
**K**	0.632 (0.470 - 0.794)	0.109	0.467 (0.248 - 0.698)	0.803 (0.695 - 0.878)	0.744	0.333	0.877
**Cl**	0.613 (0.439 - 0.786)	0.170	0.467 (0.248 - 0.698)	0.792 (0.684 - 0.869)	0.747	0.333	0.879
**Ca**	0.519 (0.348 - 0.689)	0.816	0.267 (0.109 - 0.519)	0.887 (0.793 - 0.941)	0.779	0.333	0.851
**P**	0.572 (0.393 - 0.750)	0.381	0.600 (0.357 - 0.801)	0.578 (0.461 - 0.685)	0.581	0.231	0.872
**Mg**	0.562 (0.381 - 0.742)	0.453	0.400 (0.198 - 0.642)	0.831 (0.727 - 0.900)	0.756	0.333	0.868
**Fe**	0.533 (0.371 - 0.695)	0.686	0.867 (0.621 - 0.976)	0.352 (0.251 - 0.468)	0.442	0.220	0.926
**A:G**	0.532 (0.389 - 0.673)	0.657	0.150 (0.052 - 0.360)	0.980 (0.928 - 0.996)	0.839	0.600	0.850
**DB:TB**	0.559 (0.364 - 0.753)	0.506	0.571 (0.325 - 0.786)	0.667 (0.525 - 0.783)	0.625	0.333	0.800
**URE:CREA**	0.587 (0.423 - 0.751)	0.267	0.375 (0.184 - 0.613)	0.888 (0.805 - 0.937)	0.810	0.375	0.888
**CREA:URE**	0.587 (0.423 - 0.751)	0.267	0.750 (0.409 - 0.955)	0.584 (0.480 - 0.681)	0.810	0.375	0.888
**URE:ALB**	0.588 (0.435 - 0.739)	0.267	0.938 (0.716 - 0.996)	0.241 (0.163 - 0.341)	0.340	0.183	0.952
**LDH:ALB**	0.512 (0.339 - 0.685)	0.876	0.500 (0.280 - 0.720)	0.651 (0.545 - 0.743)	0.624	0.189	0.875

ACC, accuracy; A:G, albumin to globulin ratio; ALB, albumin; ALP, alkaline phosphatase; AST, aspartate aminotransferase; AUC, area under curve; BA, bile acids; URE:ALB, urea to albumin; URE:CREA, urea to creatinine ratio; Ca, total calcium; CK, creatine kinase; Cl, chloride; CI, confidence interval; CREA, creatinine; CREA:URE, creatinine to urea ratio; DB, direct bilirubin; DB:TB, direct bilirubin to total bilirubin; EP, equine piroplasmosis; Fe, iron; FIB, fibrinogen; GGT, gamma-glutamyl transferase; GLDH, glutamate dehydrogenase; GLO, globulin; GLU, glucose; IB, indirect bilirubin; K, potassium; LDH, lactate dehydrogenase; LDH:ALB, LDH to albumin; Mg, total magnesium; Na, sodium; NPV, negative predictive value; P, phosphorus; PPV, positive predictive value; SDMA, symmetric dimethylarginine; TB, total bilirubin; TGL, triglycerides; TP, total proteins; SEN, sensitivity; SPE, specificity; URE, urea.
